# Food insecurity, drug resistance and non-disclosure are associated with virologic non-suppression among HIV pregnant women on antiretroviral treatment

**DOI:** 10.1371/journal.pone.0256249

**Published:** 2021-08-18

**Authors:** Bhavna H. Chohan, Keshet Ronen, Brian Khasimwa, Daniel Matemo, Lusi Osborn, Jennifer A. Unger, Alison L. Drake, Ingrid A. Beck, Lisa M. Frenkel, John Kinuthia, Grace John-Stewart

**Affiliations:** 1 Department of Global Health, University of Washington, Seattle, Washington, United States of America; 2 Center for Virus Research, Kenya Medical Research Institute, Nairobi, Kenya; 3 Department of Pediatrics, University of Nairobi, Nairobi, Kenya; 4 Department of Research and Programs, Kenyatta National Hospital, Nairobi, Kenya; 5 Department of Obstetrics and Gynecology, University of Washington, Seattle, Washington, United States of America; 6 Center for Infectious Diseases Research, Seattle Children’s Research Institute, Seattle, Washington, United States of America; 7 Department of Medicine, University of Washington, Seattle, Washington, United States of America; 8 Department of Pediatrics, University of Washington, Seattle, Washington, United States of America; 9 Department of Laboratory Medicine, University of Washington, Seattle, Washington, United States of America; 10 Department of Epidemiology, University of Washington, Seattle, Washington, United States of America; Fred Hutchinson Cancer Research Center, UNITED STATES

## Abstract

We determined social and behavioral factors associated with virologic non-suppression among pregnant women receiving Option B+ antiretroviral treatment (ART). Baseline data was used from women in Mobile WAChX trial from 6 public maternal child health (MCH) clinics in Kenya. Virologic non-suppression was defined as HIV viral load (VL) ≥1000 copies/ml. Antiretroviral resistance testing was performed using oligonucleotide ligation (OLA) assay. ART adherence information, motivation and behavioral skills were assessed using Lifewindows IMB tool, depression using PHQ-9, and food insecurity with the Household Food Insecurity Access Scale. Correlates of virologic non-suppression were assessed using Poisson regression. Among 470 pregnant women on ART ≥4 months, 57 (12.1%) had virologic non-suppression, of whom 65% had HIV drug resistance mutations. In univariate analyses, risk of virologic non-suppression was associated with moderate-to-severe food insecurity (RR 1.80 [95% CI 1.06–3.05]), and varied significantly by clinic site (range 2%-22%, p <0.001). In contrast, disclosure (RR 0.36 [95% CI 0.17–0.78]) and having higher adherence skills (RR 0.70 [95% CI 0.58–0.85]) were associated with lower risk of virologic non-suppression. In multivariate analysis adjusting for clinic site, disclosure, depression symptoms, adherence behavior skills and food insecurity, disclosure and food insecurity remained associated with virologic non-suppression. Age, side-effects, social support, physical or emotional abuse, and distance were not associated with virologic non-suppression. Prevalence of virologic non-suppression among pregnant women on ART was appreciable and associated with food insecurity, disclosure and frequent drug resistance. HIV VL and resistance monitoring, and tailored counseling addressing food security and disclosure, may improve virologic suppression in pregnancy.

## Introduction

Prevention of mother-to-child HIV transmission (PMTCT) programs currently reach >90% of pregnant women in regions of sub-Saharan Africa (SSA) [1]. While high antiretroviral treatment (ART) coverage is encouraging, poor ART adherence in pregnancy and postpartum remains a challenge. Poor ART adherence in pregnancy can lead to viral non-suppression and HIV drug resistance, increasing risk of maternal treatment failure and MTCT [2, 3]. Prevalence of drug resistance among pregnant or postpartum women living with HIV (WLWH) in SSA has ranged from 6% to 46% [4–7].

Adherence to ART may falter during pregnancy or postpartum due to varied factors, including stigma, non-disclosure, partner violence, and side-effects [2, 8–12]. Food insecurity has been associated with virologic non-suppression in adults and with maternal stress in the peripartum period [13–17]. It is also important to understand women’s motivation, knowledge, and behavioral skills to sustain ART adherence in pregnancy.

## Materials and methods

### Study population

This post-hoc analysis used enrollment data from a 3-arm randomized controlled trial (RCT) of short message service (SMS) in PMTCT (Mobile WAChX, NCT02400671), conducted at 6 public, Ministry of Health MCH clinics in Kenya. Women were eligible if they were ≥14 years old, attending antenatal care (ANC), HIV-infected, and had daily access to a mobile phone. The design and methods of the Mobile WAChX study have been described [18]. In brief, pregnant WLWH were enrolled between November 2015 and May 2017, and randomized to 1-way SMS, 2-way SMS, or control (no SMS). A questionnaire was administered to ascertain ART adherence information, motivation and behavioral skills (IMB) using an abbreviated LifeWindows instrument [19], social support using the Medical Outcomes Study (MOS) survey [20], partner abuse using the Abuse Assessment Screen [21], and depressive symptoms using the Patient Health Questionnaire-9 (PHQ-9) [22]. Stigma was assessed using the 4-question version of the Stigma Scale for Chronic Illness (SSCI) [23]. Food security was assessed using the Household Food Insecurity Access Scale (HFIAS) [16, 24]. ART initiation date and regimen were abstracted from clinic records. Participant blood samples were collected to perform ARV resistance analyses and HIV VL test if required. A survey was conducted to assess facility PMTCT services to examine if any services or clinic characteristics were associated with virologic non-suppression among the women. Follow-up of Mobile WAChX participants lasted 24 months, with the final study visit conducted in January 2020. This manuscript presents analysis of enrollment data only.

#### Virologic analyses

At enrolment, consent was obtained and participant VL was abstracted from program data. If program VL was unavailable, VL testing of study samples was conducted at the Kenya Medical Research Institute (KEMRI)/Centers for Disease Control and Prevention (CDC) in Kisumu or Nairobi, Kenya using the Roche COBAS® TaqMan® Analyzer or COBAS® TaqMan® Version 2.0 (CAP/CTMv2.0) platform with a lower limit of quantification of 40 copies/ml.

#### ARV resistance analyses

ARV drug resistance mutations were identified in cryopreserved plasma samples from women with HIV RNA levels ≥ 1000 copies/mL using an oligonucleotide ligation assay (OLA) [25]. The OLA assay detects mutations at HIV-1 *po*l reverse transcriptase codons 65, 103, 181, 184, and 190 that can confer resistance to non-nucleoside reverse transcriptase inhibitors (NNRTI) and nucleoside/tide reverse transcriptase inhibitors (NRTI). OLA probes have been optimized for HIV subtypes A, D, and C, common in Kenya [25]. Viral RNA extracted from plasma was reverse-transcribed and resultant cDNA was analyzed using OLA and consensus sequencing when OLA testing failed at any of the codons analyzed [25]. The study classified resistance mutations as detected if they were at an abundance of 10% or more.

#### Statistical methods and data analysis

Statistical analysis was performed using Stata version 13 [26]. PHQ-9 scores were dichotomized with score ≥ 5 indicating at least mild depressive symptoms. HFIAS scores classified food insecurity into four levels per instrument guidelines: food secure, mildly, moderately, or severely food insecure and dichotomized into secure/mildly insecure vs. moderate/severely insecure [24]. Virologic non-suppression was defined as VL ≥ 1000 copies/ml. Analysis was restricted to women on ART for ≥ 4 months at enrollment, to allow sufficient time to achieve virologic suppression. Correlates of virologic non-suppression were determined by Poisson regression with robust standard errors. Multivariate analysis was conducted including all variables that were associated with virologic non-suppression in univariate analysis at a significance level of p<0.1.

#### Ethical review

Ethical approval was obtained from the University of Washington and Kenyatta National Hospital/University of Nairobi institutional review boards. The study was performed in accordance with ethical standards in the 1964 Declaration of Helsinki and its later amendments or comparable ethical standards. Participants provided written informed consent prior to enrollment.

## Results

### Study population

Among 824 participants, 470 women had been on ART for ≥ 4 months at enrollment and were included in analyses. Median age was 28 years, the majority (86.0%) of women were married or cohabiting, and 9.2% were primigravida. Overall, 122 (26.0%) women reported at least mild depressive symptoms (PHQ9 score ≥ 5). There was a high rate of HIV status disclosure with 96.6% women reporting disclosure to anyone and 90.1% reporting disclosure to their partner. The majority (61.9%) of women were on efavirenz-based ART. Of the 470 women included in the analysis, 429 (91.3%) VL results were evaluated by the study from study plasma specimens, while 41 (8.7%) were obtained from clinical record abstraction. Of these 470 women, 57 (12.1%) women had unsuppressed viral load (VL ≥ 1000 copies/ml).

### HIV drug resistance in women with non-suppressed VL

Among 57 women with virologic non-suppression, 54 were tested for resistance. Thirty-five (64.8%) had detectable mutations conferring resistance to NNRTIs and NRTIs (S1 Fig), of whom 32 were on NNRTIs (15 on TDF/3TC/EFV, 9 on TDF/3TC/NVP, 8 on ZDV/3TC/NVP), and 3 were on PI-based ART (TDF/3TC/LPV/r or ZDV/3TC/LPV/r or ZDV/3TC/ATV/r).

Among women on NNRTI-based ART, single resistance mutations detected were: K103N only (n = 5), Y181C only (n = 2), G190A only (n = 2), M184V only (n = 1); dual mutations: K103N and M184V (n = 11), G190A and M184V (n = 3), Y81C and M184V (n = 2); and multiple mutations: K103N, G190A and M184V (n = 2); Y181C, G190A and M184V (n = 1); Y181C, K65R and M184V (n = 3); K103N + K65R + M184V (n = 1); G190A + K65R + M184V (n = 1); and G190A + K101H + M184V (n = 1) (S1 Fig). We detected G190A, a NNRTI mutation in 2 women on LPV/r-based ART, and a K103N mutation in one woman on ATV/r-based ART, suggesting either transmitted drug resistance or prior exposure to NNRTI-based ARVs among 3 women on PI-based ART.

### Correlates of virologic non-suppression

HIV status disclosure was associated with lower risk of virologic non-suppression: 11.3% women who had disclosed were unsuppressed compared with 31.3% of those who had not disclosed (RR 0.36 [95% CI 0.17–0.78]) (Table 1). Higher IMB scores were associated with lower risk of virologic non-suppression (RR 0.67 [95% CI 0.46–0.98] per 10% increase in IMB score). In analyses of IMB components, only behavior skills were associated with lower risk of non-suppression (RR 0.70 [95% CI 0.58–0.85]). Moderate-to-severe food insecurity was also associated with virologic non-suppression: 15.2% of food-insecure women had unsuppressed VL compared with 8.5% of food-secure women (RR 1.80 [95% CI 1.06–3.05]). Marital status, self-reported ART adherence, history of side effects, social support, history of partner abuse or distance from home to clinic were not associated with virologic non-suppression. In multivariate analysis adjusted for all variables associated with virologic non-suppression in univariate analysis, virologic non-suppression remained significantly associated with food insecurity (aRR 2.07 [95% CI 1.15–3.72]) and status disclosure (aRR 0.34 [95% CI 0.15–0.78]) (Table 1). Differences by clinic also remained statistically significant.

**Table 1:** Correlates of unsuppressed VL (among those on ART ≥ 4 months).

	Suppressed VL (<1000 c/ml)	Unsuppressed VL (≥1000 c/ml)	Univariate analysis		Multivariate analysis	
	n(%) or median (IQR)		RR (95% CI)	p-value	aRR^+^ (95% CI)	p-value
Overall	413 (87.9%)	57 (12.1%)				
Age (years)	28 (24–32)	28 (25–31)	0.98 (0.95–1.03)	0.47		
Marital status			0.61 (0.34–1.10)	0.10		
Married/cohabiting	359 (88.9)	45 (11.1)				
Unmarried & not cohabiting	54 (81.8)	12 (18.2)				
Self-reported adherence on VAS			1.54 (0.87–2.71)	0.14		
<95% on VAS	74 (84.1)	14 (15.9)				
≥95% on VAS	338 (89.7)	39 (10.3)				
ART doses missed in last 30 days			0.79 (0.33–1.90)	0.59		
>0	50 (90.9)	5 (9.1)				
0	352 (88.4)	46 (11.6)				
Self-reported adherence			0.98 (0.58–1.65)	0.94		
<”excellent”	188 (89.1)	23 (10.9)				
“excellent”	224 (88.9)	28 (11.1)				
Regimen (self-report)						
TDF/3TC/EFV	260 (89.4)	31 (10.7)	Ref			
TDF/3TC/NVP	85 (85.9)	14 (14.1)	1.33 (0.74–2.39)	0.35		
ZDV/3TC/NVP	48 (85.7)	8 (14.3)	1.34 (0.65–2.76)	0.43		
TDF/3TC/LPV/r or ZDV/3TC/LPV/r	14 (87.5)	2 (12.5)	1.17 (0.31–4.48)	0.82		
Other	6 (75.0)	2 (25.0)	2.35 (0.67–8.17)	0.18		
History of regimen switch			1.54 (0.60–3.94)	0.37		
Yes	21 (84.0)	4 (16.0)				
No	387 (89.6)	45 (10.4)				
Any side effects in last 30 days			0.80 (0.44–1.45)	0.47		
Yes	121 (90.3)	13 (9.7)				
No	291 (87.9)	40 (12.1)				
ART adherence IMB %*	77.3 (73.3–82.7)	76.7 (70.0–82.0)	0.67 (0.46–0.98)	**0.04**	0.88 (0.69–1.13)	0.32
Information score %*	85.0 (80.0–95.0)	85.0 (75.0–100.0)	0.96 (0.78–1.17)	0.66		
Motivation score %*	55.0 (40.0–75.0)	60.0 (40.0–75.0)	1.01 (0.89–1.15)	0.88		
Behavior skills score %*	82.9 (77.1–91.4)	80.0 (74.3–85.7)	0.70 (0.58–0.85)	**<0.001**		
Depression symptoms			1.54 (0.93–2.55)	0.09	1.12 (0.57–2.18)	0.74
≥mild (≥5 PHQ9)	102 (83.6)	20 (16.4)				
<mild (<5 PHQ9)	311 (89.4)	37 (10.6)				
Social support score*	88.9 (69.4–100.0)	87.5 (75.0–100.0)	1.04 (0.93–1.17)	0.51		
Food security			1.80 (1.06–3.05)	**0.03**	2.07 (1.15–3.72)	**0.02**
Moderate/severe food insecurity	218 (84.8)	39 (15.2)				
≤Mild food insecurity	195 (91.6)	18 (8.5)				
Time to travel to clinic (minutes)	30.0 (20.0–60.0)	30.0 (22.5–60.0)	1.00 (0.99–1.01)	0.45		
Status disclosure to partner			0.60 (0.30–1.20)	0.15		
Yes	357 (89.0)	44 (11.0)				
No	36 (81.8)	8 (18.2)				
Status disclosure to anyone			0.36 (0.17–0.78)	**0.01**	0.34 (0.15–0.78)	**0.01**
Yes	401 (88.7)	51 (11.3)				
No	11 (68.8)	5 (31.3)				
Any stigma			0.99 (0.49–2.01)	0.99		
Yes	85 (88.5)	11 (11.5)				
No	138 (88.5)	18 (11.5)				
History of abuse			1.02 (0.55–1.90)	0.94		
Ever	78 (87.6)	11 (12.4)				
Never	335 (87.9)	46 (12.1)				
Clinics						
Nairobi 1	71 (78.0)	20 (22.0)	Ref		Ref	
Nairobi 2	52 (98.1)	1 (1.9)	0.09 (0.01–0.62)	**0.02**	0.09 (0.01–0.65)	**0.02**
Western 1	105 (92.9)	8 (7.1)	0.32 (0.15–0.70)	**0.004**	0.34 (0.14–0.80)	**0.01**
Western 2	95 (91.4)	9 (8.7)	0.35 (0.16–0.76)	**0.008**	0.43 (0.19–0.94)	**0.03**
Western 3	32 (88.9)	4 (11.1)	0.51 (0.19–1.38)	0.18	0.42 (0.15–1.16)	0.10
Western 4	55 (75.3)	18 (24.7)	1.00 (0.56–1.78)	0.99	0.68 (0.29–1.56)	0.36

Key

*estimate for 10% increase in score

^+^ includes all variables associated with non-suppression in univariate analysis Ref = reference

RR = Relative risk; per unit increase for continuous variables

IQR = Interquartile Range

VAS = Visual analog scale on ART adherence

IMB = ART adherence information, motivation and behavioral skills scores. The scores, ascertained using the LifeWindows questionnaire, indicate participants’ level of information (knowledge) about ART adherence, motivation to adhere, and behavioral skills to adhere.

ART = Antiretroviral therapy

ART regimen: TDF = Tenofovir; 3TC = Lamivudine; ZDV = Zidovudine; EFV = Efavirenz; NVP = Nevirapine; LPV/r = Ritonavir boosted Lopinavir.

Prevalence of virologic non-suppression was significantly higher at 2 clinics (one in Nairobi, 22%; one in Western Kenya, 25%), compared with other clinics (prevalence 2–11%) (Table 1). We did not detect significant differences between facilities in staffing levels, experience dispensing ART, ART stockouts, practices regarding VL testing, or support provided to patients with non-suppression (S1 Table). There was a higher prevalence of depression (p <0.001) and lower median IMB scores (p <0.001) among study participants in facilities with a higher prevalence of virologic non-suppression (>20%) (S2 Table).

## Discussion

In this study of pregnant women on ART for ≥ 4 months attending urban and rural clinics in Kenya, 12% had unsuppressed HIV VL, 65% of whom had resistance mutations. Women who had not disclosed their HIV status, had food insecurity, and had poorer adherence behavioral skills were more likely to have virologic non-suppression. In multivariate analysis, status disclosure and food insecurity were independently associated with virologic non-suppression. Prevalence of virologic non-suppression varied by clinic; however, there were no discernable facility characteristics associated with non-suppression.

Our findings are consistent with other studies from SSA, which report 6–20% prevalence of virologic non-suppression among women on Option B+ [6, 27–30]. However, most of these studies evaluated VL postpartum following ART initiation in pregnancy, in contrast to our study which focused on pregnancy. The majority of women entering PMTCT programs in SSA are already on ART [31]. Our study suggests that assessing VL among pregnant women already on ART remains important.

Among women with unsuppressed VL in our study, 65% had detectable resistance mutations, mainly to NNRTIs. Rates of drug resistance among women on Option B+ in SSA have ranged from 6% to 46% in smaller studies [4, 5]. Our findings suggest a need for resistance testing among pregnant women receiving NNRTI regimens. With increased use of dolutegravir, rates of resistance are expected to decrease [32, 33].

We used the LifeWindows IMB ART Adherence Questionnaire, which evaluates domains that influence ART adherence based on the IMB behavioral theory–information, motivation and behavioral skills [34, 35]. Women with lower scores were more likely to have virologic non-suppression and this association was driven by differences in behavior skills rather than motivation or information. To our knowledge, this is the first evaluation of the IMB construct in PMTCT programs. Rapid assessment of behavioral skills using this instrument could be used to identify clients who need support. Although this association was not independently significant when adjusting for other correlates, univariate association of this easily assessed construct is potentially clinically meaningful and actionable.

Women in our study who had disclosed their HIV status were less likely to have virologic non-suppression, consistent with other studies [36]. Women with virologic non-suppression had higher prevalence of moderate or severe household food insecurity, consistent with studies in other populations [13–15, 37]. To our knowledge, this is the first study assessing food insecurity and virologic non-suppression in PMTCT and suggests that food supplementation or agricultural support may optimize both maternal-child nutrition and viral suppression. Our findings suggest that wholistic care that addresses depression, disclosure and food security could help women adhere to ART.

We found wide variation in prevalence of virologic non-suppression between clinics; however, there were no discernable differences in facility characteristics. Women attending clinics with a high prevalence of virologic non-suppression had significantly lower ART adherence behavior skills scores and higher prevalence of at least mild depression than women at the other clinics, which suggests participant rather than facility factors explained differences in virologic non-suppression prevalence between clinics. Because behavior scores varied by clinic, clinic-adjusted analyses may have adjusted away these associations. Thus, understanding both overall and site-adjusted cofactors is important in our study.

## Conclusions

In summary, we found that virologic non-suppression among pregnant WLWH on ART was more frequent among women who had not disclosed their HIV status, were food insecure, or had lower adherence behavioral skills. Addressing these factors may improve viral suppression among pregnant women. Peer navigators, mentor mothers, or interventions to increase male partner involvement may be beneficial [36]. Our resistance data suggest that non-suppression was often due to resistance. While prevalence of resistance will decrease as PMTCT programs move to dolutegravir use, women on NNRTI regimens would benefit from resistance testing. Assessing behavioral skills with the Lifewindows IMB instrument may be useful to prioritize women for adherence interventions and to monitor intervention effects in PMTCT programs.

## Supporting information

S1 Fig Schematic representation of HIV drug resistance in study women with unsuppressed plasma viral load. Of the 470 pregnant women living with HIV on ART for ≥4 months, 57 (12.1%) had unsuppressed plasma viral load (HIV viral load ≥1000) at enrollment. Of these, blood samples were available for 54 women, and of those 35 women (64.8%) had detectable drug resistance mutations mainly to NNRTI antiretrovirals. The majority of the women with detectable HIV drug resistance mutations, as assessed by >10% level using oligonucleotide assay (OLA) and consensus sequencing, were on 1^st^ line NNRTI-based ART, except for three women on ^$^Protease-inhibitor based ART had NNRTI drug resistance mutation. NNRTI, nonnucleoside reverse transcriptase inhibitor; NRTI, nucleoside reverse transcriptase inhibitor; ART (antiretroviral) regimen: TDF = tenofovir; 3TC = lamivudine; ZDV = Zidovudine; EFV = efavirenz; NVP = nevirapine; LPV/r = ritonavir-boosted lopinavir; *TAMs = Thymidine analogue mutations (*1 TAM (n = 1), **2 TAMs (n = 2)).
(DOCX)

S1 Table Differences in facility characteristics. (DOCX)

S2 Table Differences in individual-level characteristics by site. (DOCX)
