# Supplementary material for: Food insecurity, drug resistance and non-disclosure are associated with virologic non-suppression among HIV pregnant women on antiretroviral treatment
Source: PLoS One. 2021 Aug 18;16(8):e0256249. doi: 10.1371/journal.pone.0256249 (PMC8372899; doi:10.1371/journal.pone.0256249)
Supplement: S1 Fig — Of the 470 pregnant women living with HIV on ART for ≥4 months, 57 (12.1%) had unsuppressed plasma viral load (HIV viral load ≥1000) at enrollment. Of these, blood samples were available for 54 women, and of those 35 women (64.8%) had detectable drug resistance mutations mainly to NNRTI antiretrovirals. The majority of the women with detectable HIV drug resistance mutations, as assessed by >10% level using oligonucleotide assay (OLA) and consensus sequencing, were on 1st line NNRTI-based ART, except for three women on $Protease-inhibitor based ART had NNRTI drug resistance mutation. NNRTI, nonnucleoside reverse transcriptase inhibitor; NRTI, nucleoside reverse transcriptase inhibitor; ART (antiretroviral) regimen: TDF = tenofovir; 3TC = lamivudine; ZDV = Zidovudine; EFV = efavirenz; NVP = nevirapine; LPV/r = ritonavir-boosted lopinavir; *TAMs = Thymidine analogue mutations (*1 TAM (n = 1), **2 TAMs (n = 2)). (DOCX) [file pone.0256249.s001.docx]

## **S1 Fig: Schematic representation of HIV drug resistance in study women with unsuppressed plasma viral load**

Participants N=57

baseline resistance testing with VL ≥1000 c/ml

Resistance detected N=35

Mutations

1. NNRTI only (n=9):

K013N (n=5)^$^, G190A (n=2)^$^,

Y181C (n=2)

2. NRTI only (n=1):

M184V (n=1)

3. NNRTI and NRTI (n=25):

K103N + M184V** (n=11)

G190A + M184V* (n=3)^$^

Y181C + M184V (n=2)

K103N + G190A + M184V (n=2)

Y181C + G190A + M184V (n=1)

Y81C + K65R + M184V* (n=3)

K103N + K65R + M184V (n=1)

G190A + K65R + M184V (n=1)

G190A + K101H + M184V (n=1)

ART Regimen

TDF/3TC/EFV (n=15)

TDF/3TC/NVP n=9)

ZDV/3TC/NVP (n=8)

TDF/3TC/ATv/r (n=1)^$^

TDF/3TC/LPV//r (n=2) ^$^

Participants tested for resistance N=54

**S1 Fig**: **Schematic representation of HIV drug resistance in study women with non-suppression of HIV RNA plasma viral load**. Of the 470 pregnant women living with HIV on ART for ≥4 months, 57 (12.1%) had unsuppressed plasma viral load (HIV viral load ≥1000) at enrollment. Of these, blood samples were available for 54 women, and of those, 35 women (64.8%) had detectable drug resistance mutations mainly to NNRTI antiretrovirals. The majority of the women with detectable HIV drug resistance mutations, as assessed by >10% level using oligonucleotide assay (OLA) and consensus sequencing, were on 1^st^ line NNRTI-based ART, except for three women on ^$^Protease-inhibitor based ART had NNRTI drug resistance mutation.

NNRTI, nonnucleoside reverse transcriptase inhibitor; NRTI, nucleoside reverse transcriptase inhibitor; ART (antiretroviral) regimen: TDF = tenofovir; 3TC = lamivudine; ZDV = Zidovudine; EFV = efavirenz; NVP = nevirapine; LPV/r = ritonavir-boosted lopinavir; *TAMs = Thymidine analogue mutations (*1 TAM (n=1), **2 TAMs (n=2))
