# Supplementary material for: Food insecurity, drug resistance and non-disclosure are associated with virologic non-suppression among HIV pregnant women on antiretroviral treatment
Source: PLoS One. 2021 Aug 18;16(8):e0256249. doi: 10.1371/journal.pone.0256249 (PMC8372899; doi:10.1371/journal.pone.0256249)
Supplement: S1 Table — (DOCX) [file pone.0256249.s002.docx]

## **S1 Table: Differences in facility characteristics**

| **Facility (clinic) characteristics** | **Nairobi 1** | **Nairobi 2** | **Western 1** | **Western 2** | **Western 3** | **Western 4** |
| --- | --- | --- | --- | --- | --- | --- |
| Facility location | urban | urban | rural | rural | rural | rural |
| Facility type | Health Center | Health Center | Provincial Hospital | Provincial Hospital | Provincial Hospital | Provincial Hospital |
| NGO support | Yes | Yes | Yes | Yes | Yes | Yes |
| Approximate number of HIV-infected adults active in care | 1965 | 3211 | 3643 | 3853 | 3400 | 3721 |
| Approximate number of pregnant women currently active in ANC | 500 | 670 | Not available | 342 | 450 | 388 |
| ***Staffing at the Facility level*** | | | | | | |
| Doctors/COs | 5 | 3 | 16 | 5 | 7 | 2 |
| Nurses | 25 | 3 | 40 | 7 | 6 | 8 |
| # of Peer Counsellors | 3 | 5 | 11 | 7 | 10 | 7 |
| ***HIV Viral Load testing*** | | | | | | |
| Distance to laboratory for HIV Viral Load testing (km) | 14 | 10 | 25 | 50 | 200 | 0.2 |
| Frequency of clinic receiving VL results from lab | 7-14 days | weekly | weekly | 10-15 days | ~15 days | ~14 days |
| ***ART treatment monitoring, support and supplies*** | | | | | | |
| Are there support groups for HIV infected adults in your facility? | Yes | Yes | Yes | Yes | Yes | Yes |
| Is there a Mothers-to-Mothers group at the clinic? | Yes | No | Yes | No | No | Yes |
| Are there peer educators at the clinic? | Yes | Yes | Yes | Yes | Yes | No |
| Stock-out of ARVs in the last 3months | TDF/3TC 7days  TDF/3TC/DTG 2 days | ABC 45 days TDF/3TC 45d NVP syrup 21 days | LPV/r tabs 20 days NVP syrup 30 days | No | NVP syrup 2 months | No |
| Has stock-out of ARVs affected prescribing? | Refills given for 2 weeks & get more or borrow from other facilities | Less dose prescribed, thus more prescriptions; transfer of clients to other facilities | Substitute LPV/r tabs with ATV/r, for <40kg LPV/r syrup. Dispense AZT syrup instead of NVP syrup | N/A | NVP substituted with AZT | N/A |
| Stock-out of VL supplies in last 3m | No | No | No | No | No | No |
