# Supplementary material for: Food insecurity, drug resistance and non-disclosure are associated with virologic non-suppression among HIV pregnant women on antiretroviral treatment
Source: PLoS One. 2021 Aug 18;16(8):e0256249. doi: 10.1371/journal.pone.0256249 (PMC8372899; doi:10.1371/journal.pone.0256249)
Supplement: S2 Table — (DOCX) [file pone.0256249.s003.docx]

## **S2 Table: Differences in individual-level characteristics by site**

|  |  | “Low failure” sites (<15% prevalence, Clinics: Nairobi 2, Western 1, 2, 3) | “High failure” sites (>20% prevalence, Clinics: Nairobi 1, Western 4) | p-value* |
| --- | --- | --- | --- | --- |
|  | N | n(%) or median (IQR) | |  |
| Overall | 470 | 306 (65.1) | 164 (34.9) |  |
| Age (years) | 470 | 28.5 (24-32) | 28 (24-31) | 0.21 |
| Age category  <25  25-34  ≥35 | 129  276  65 | 84 (27.5)  175 (57.2)  47 (15.4) | 45 (27.4)  101 (61.6)  18 (11.0) | 0.40 |
| ART IMB behavior score % | 406 | 82.9 (80.0-94.3) | 80.0 (74.3-85.7) | **<0.001** |
| ART IMB information score % | 444 | 90.0 (80.0-95.0) | 80.0 (75.0-100.0) | 0.19 |
| ART IMB motivation score % | 445 | 50.0 (40.0-70.0) | 65.0 (40.0-80.0) | **<0.001** |
| ART IMB composite score % | 387 | 77.3 (74.7-82.7) | 77.3 (72.0-82.7) | 0.06 |
| At least mild depression (>=5 PHQ9) | 470 | 55 (18.0) | 67 (40.9) | **<0.001** |
| Status disclosure to anyone | 468 | 297 (97.4) | 155 (95.1) | 0.20 |

*χ^2^ for categorical, t-test for continuous variable
